# Primary malignant mucosal melanoma of the upper lip: a case report and review of the literature

**DOI:** 10.1186/s13104-015-1459-3

**Published:** 2015-09-29

**Authors:** Narayan Sharma Lamichhane, Jiping An, Qilin Liu, Wei Zhang

**Affiliations:** Department of Oral and Maxillofacial Surgery, Norman Bethune Hospital of Stomatology, Jilin University, Qinghua Road, Changchun, 130021 Jilin People’s Republic of China; Department of Oral and Maxillofacial Surgery, School of Stomatology, Jiamusi University, Xuefu Street, Jiamusi, 15400 Heilongjiang China

**Keywords:** Biopsy, Malignant melanoma, Oral melanoma, Prognosis

## Abstract

**Background:**

Malignant melanoma of oral cavity is a rare condition, accounting for 0.5 % of all oral malignancies and about 1–2 % of all melanomas. Oral melanomas have extremely poor prognosis with 5 years survival rate of 12.3 %. The poor prognosis compared to cutaneous melanoma may be attributed to delay in reporting by patient and diagnosis, and apt to become ulcerated due to repeated trauma. The ‘chameleonic’ presentation of a mainly asymptomatic condition, the rarity of these lesions, the poor prognosis and the necessity of a highly specialized treatment are factors that should be seriously considered by the involved health provider.

**Case presentation:**

We present a case of 32 years old male of Han ethnicity with mucosal melanoma of upper lip, comparing his clinical presentation and histological findings at his first visit and following the recurrence. The patient complained of black discoloration on the left side of upper lip since 4 years which gradually increased in size and later involved the skin of the lip. Excision with 5 mm safety margin was performed but the patient presented with the similar lesion after three and half years of the treatment. So, again wide excision with 2 cm safety margin was performed followed by reconstruction of the lip.

**Conclusion:**

This case provides an example of aggressive behavior of mucosal melanoma and emphasizes on the fact that any pigmented lesion detected in the oral cavity may exhibit potential growth and should be submitted to biopsy to exclude malignancy. It also exemplifies of how the time of diagnosis and the evolution of a disease could be seriously influenced by patient’s behavior.

## Background

Malignant melanoma is a malignant neoplasm of melanocytes or of melanocytic precursors [1]. Primary mucosal melanomas of the head and neck are rarer than cutaneous melanoma. Among those of the head and neck region, oral mucosal melanoma is extremely infrequent accounting for only 0.5 % of oral neoplasms and 1–2 % of all melanomas. Primary oral melanoma is a rare neoplasm arising from uncontrolled growth of melanocytes found in the basal layers of oral mucosal epithelium. Melanocytes are derived from neural crest cells and migrate to several sites, including skin and mucous membrane [2]. Oral mucosal melanomas are highly malignant tumors with the tendency to metastasize or locally invade tissues more readily than other malignant tumors of the oral cavity [3]. Hard palate and maxillary gingiva are the common sites for oral melanoma. Oral melanoma can present as an unevenly shaped macule, plaque or mass, well circumscribed or diffused.

A review of literature revealed fewer than 50 reported cases of primary malignant melanoma of the lip. Literature review till 1997 showed 30 reported cases of mucosal melanoma of the lip [4]. The scattered data on mucosal melanoma of lip after 1997 shows less than 20 cases of mucosal melanoma of lip. A paucity of data elucidating the predictive factors as well as the unpredictable and aggressive biologic behavior of mucosal melanoma compounds the vexing clinical scenario (Table 1).

**Table 1:** Summary of literature review on mucosal melanoma

SN	Author/date	Evidence type	Sample size	Age/sex	Place of study	Relevant finding
1.	Ullah et al. (2010)	Case report	1	55 years/M	Egypt	Therapeutic neck dissection is to be done in cases of palpable lymph nodes but there is disagreement over neck dissection be done in absence of clinically palpable nodes
2.	Lourenco et al. (2009)	Case series	35	–	South America	Most cases (71.42 %) were found in hard palate and upper alveolar ridge
3.	Hajar-Serviansky et al. (2012)	Case report	1	40 years/M	Mexico	After complete removal, 10–20 % regional relapses have been reported with a 10–25 % 5 years survival rate
4	Sta`rek et al. (2006)	Pilot study	2	–	–	The presence of microscopic metastatic focus in the sentinel lymph node was associated with an early hematogenous dissemination. Therefore, sentinel lymph node biopsy, which represents a potentially efficient staging tool, warrants further investigation
5.	Govindarajan et al. (2003)	Prospective study on nude mice	–	–	–	Activation of MAP kinase signaling may be an important pathway involved in melanoma transformation. Inhibition of MAP kinase signaling may be useful in the prevention and treatment of melanoma
6.	Ali and Ali (2007)	Review article	–	–	–	*c*-*kit* signaling is believed to play a role in tumorigenesis
7	Buery et al. (2011)	Prospective study	18	–	–	RAS is intensely expressed in both in situ and invasive OMMs
8	Goldinger et al. (2013)	Review article	–	–	Switzerland	KIT mutations are found at low frequencies (≤10 %) in melanomas arising from mucosal or acral lentiginous surfaces
9.	Flaherty et al. (2012)					The combination of the BRAF inhibitor dabrafenib and the MEK inhibitor trametinib in patients with metastatic BRAF V600 melanoma, represents one strategy for delaying the emergence of this resistance mechanism (median duration of response for combination therapy 10.5 vs 5.6 months for dabrafenib monotherapy)
10.	Lian and Guo (2014)	Review article			China	Monoclonal antibodies targeting immune checkpoint proteins (include Ipilimumab and PD-1/PD-L1 antibodies) have elicited long-lasting anti-cancer response in metastatic melanoma, randomized clinical trials on checkpoint inhibitors in patients with metastatic MM are limited
11.	Garzino-Demo et al. (2004)	Case series	10	–	Italy	Oral mucosal melanomas are highly malignant tumors with the tendency to metastasize or locally invade tissues more readily than other malignant tumors of the oral cavity
12.	Manolidis and Donald (1997)	Retrospectives study and review	14	–	USA	30 reported cases of mucosal melanoma of lip till 1997
13.	Tacastacas et al. (2014)	Review article	–	–	–	A review of literature reports that 14 % of mucosal melanomas harbor activating c-KIT mutations
14.	Wang et al. (2012)	Retrospective study	61	–	China	Elective node dissection and adjuvant biochemotherapy offer no additional advantage in increasing the patient survival rate for patients with clinical stage N0 OMM
15.	Hodi et al. (2010)	Clinical trial	676	–	13 countries in North America, South America, Europe, and Africa	Ipilimumab improved overall survival in patients with previously treated metastatic melanoma
16.	Mary E. Keir et al. (2008)	Prospective study	–	–	USA	The interaction of PD-1 with its two ligands, B7-H1 and B7-DC (PD-L1 and PD-L2), occurs predominantly in peripheral tissues including the tumor microenvironment and leads to apoptosis and downregulation of T-cell effector function
17.	Lennartsson et al. (2005)	Review article	–	–	USA	C-KIT is a key regulator of growth, differentiation, migration, and proliferation of melanocytes
18.	Chaudhry et al. (1958)	Review of 105 cases	105	–	USA	80 % (93 out of 105 pts) cases of oral melanoma originated in maxilla

This case presents the aggressive nature of malignant mucosal melanoma in 32 years old male and highlights the need of careful scrutinization of any pigmented lesions in oral cavity and necessity of biopsying lesions with potential growth to rule out malignancy.

## Case presentation

A 32 years old male of Han ethic group from northeast china, driver by occupation, presented to Department of Oral and Maxillofacial Surgery, Norman Bethune Hospital of Stomatology, ChangChun, China with pigmented lesions on the upper left lip vermilion during month of June 2011. The lesion was noticed for the first time 4 years before as a macule of approximately 1 cm × 0.5 cm which gradually increased in size without any accompanying symptoms and discomfort. He also noticed a change in the color of skin of lip above left vermilion after about 3 years of the changes in the mucosa of the lip.

There is no significant past medical and family history. The patient’s family history of any other tumor was also inquired but nothing significant was reported. He has neither the history of smoking nor drinking.

**Fig. 1:**
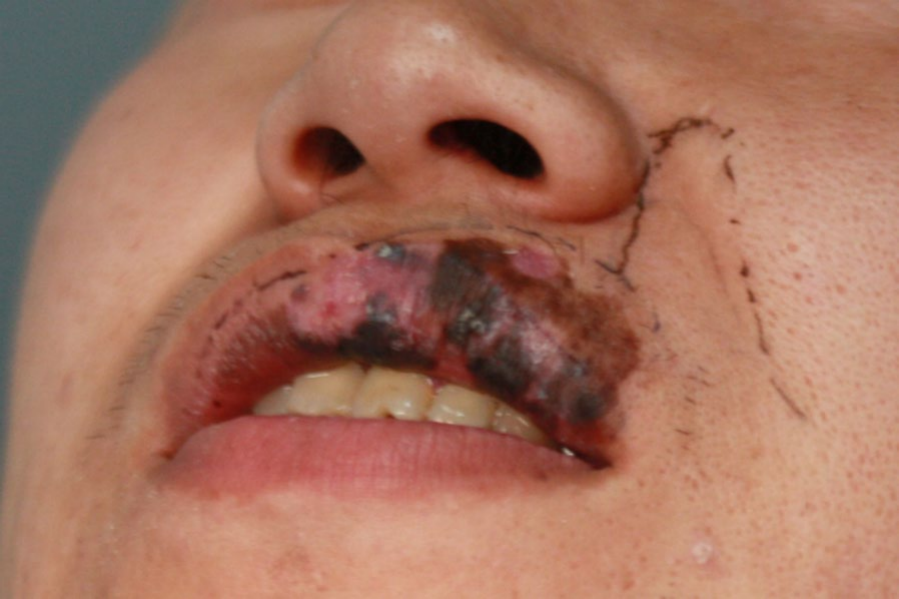
Mucosal melanoma of lip showing pigmented papules involving adjacent skin. Nasolabial flap design for reconstruction

On extra oral examination, pigmented papules of various sizes were seen in the vermilion of upper left lip associated with brownish discoloration of the skin above vermilion. The skin discoloration was approximately 0.5 × 1 cm extending from 0.5 cm medial to left angle of mouth to the left philtral ridge (Fig. 1). The lip was slightly swollen. On intra-oral examination, the mucosal lesion was about 1.5 cm above the vestibule extending from labial frenum to 0.5 cm medial to angle of mouth on the left side. It was neither indurated nor tender on palpation. There were some areas of ulcerations in the mucosa.

There was no apparent neck swelling and cervical lymph nodes were non palpable (not enlarged). There were no similar skin pathology in other parts of body except lip.

A complete blood count, biochemical profile and urine analysis were done without significant findings. No abnormality was detected in the chest X-ray. Computed tomography (CT) scan was done to find out the extent of the lesion and to rule out local and regional lymphatic involvement.

On presentation, presumptive clinical diagnosis of mucosal melanoma (MM) was made. A punch biopsy was done and the tissue was sent for histopathological analysis. The histologic features were consistent with clinical diagnosis of oral mucosal malignant melanoma.

Medical information was provided to the patient and his family regarding the diagnosis, staging, therapeutic options and prognosis. Excisional biopsy was performed with safety margin of 5 mm and frozen section biopsy was found to be free of malignant cells. Left nasolabial flap was used for reconstruction of the lip and mucosal portion of lip was covered with biological membrane and bolster dressing was given.

On Jan 2015, after three and half years of first operation, he presented with similar lesion on the lip. On examination, a nodular bulge of 3 × 2 cm was seen. Following the recurrence, the patient presented with mucosal macules of 4 × 2 mm and 3 × 2 mm on the vermilion of the upper left lip without involvement of the skin above vermilion, in contrast to the first presentation as papules.

**Fig. 2:**
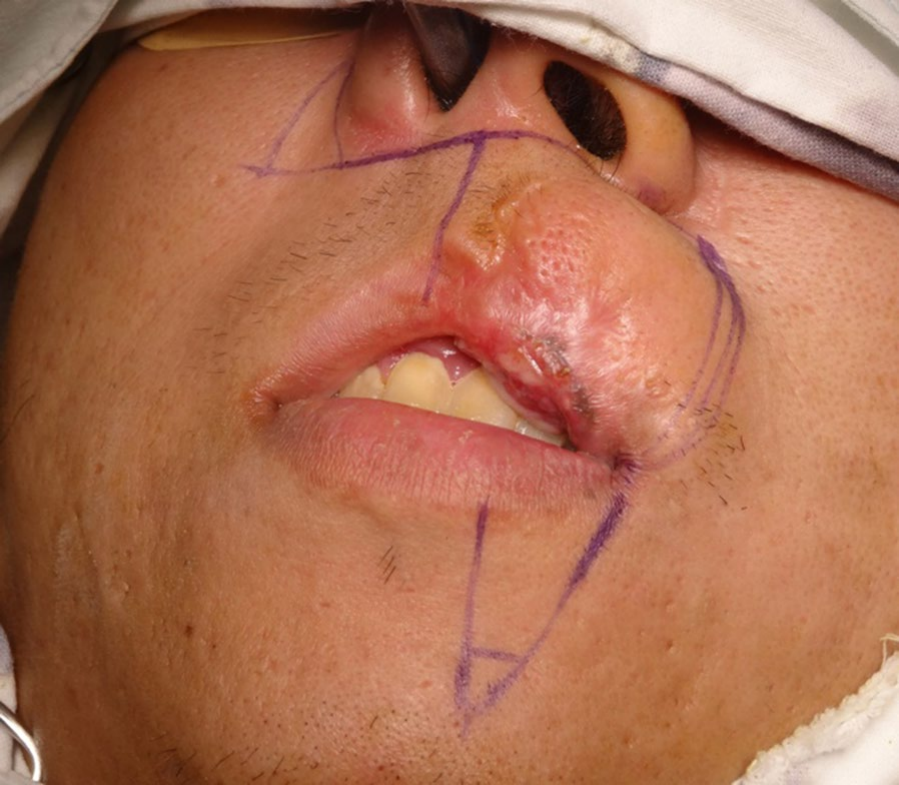
Recurrence of mucosal melanoma showing pigmented macules and a bulge on upper lip. Flap design for reconstruction

The lesion was more than 2 mm in thickness which can be correlated with stage III of Breslow’s classification although this classification has not been validated as prognostic predictors in oral melanoma due to architectural differences between oral mucosa and skin. The swelling was indurated and non-tender. There was a small area of ulceration on mucosa. The pathological report showed <1 mitoses per square millimeter. The cervical lymph nodes were not palpable. On biopsy, recurrence of the oral mucosal melanoma was confirmed. Excision of the melanoma was performed with safety margin of 2 cm in contrast to 5 mm safety margin during the first operation. Frozen section confirmed the margins to be free of malignant cells. The lip was reconstructed with Abbe-Estlander flap as well as utilizing tissue from the right nasolabial fold as shown in the Fig. 2. The patient denied of undergoing radiotherapy treatment after excision. The patient is under regular follow up and strictly advised to maintain regular follow up every 3 monthly.

## Discussion

Primary mucosal melanoma is very rare. The common sites for intraoral melanoma are the palate and maxillary gingiva accounting for 80–90 % of the cases, but any mucosal site may be involved. The other reported sites are labial and buccal mucosa, tongue and floor of the mouth. Chaudhary et al. [5], in their study of 105 cases found that 80 % (93 pts) cases of oral melanoma originated in maxilla, 51 % (38 pts) limited to hard palate, 26 % (20 pts) to alveolar ridge, 8 % (5 pts) to soft palate and 15 % (12 pts) of them belonged to more than one locations. Lourenco Martin Sangueza et al. recently reported 35 oral mucosal melanoma cases, within the Latin American population. Most cases (71.42 %) were found in hard palate and upper alveolar ridge [6]. Secondary or metastatic lesion may be located on the tongue, parotid, and tonsils [7]. In contrast to the above reported literatures, our patient presented with the melanoma on the left side of upper lip.

Most studies on melanoma reveal a higher incidence in older males showing 2:1 M:F ratio; however, Pour et al. reported a greater prevalence of oral melanoma among women [8]. The mean ages of occurrence are between the fifth and seventh decades. It is frequently found in people over 40 and seldom found in those under 20. The average age is between 51–60 years in males and 61–70 years in females [9]. Oral melanomas arise more frequently in Caucasians and Asians, with highest incidence in the Japanese [10]. Non-caucasian patients are less likely to present with cutaneous melanoma (for example Africans, due to greater level of melanin than Caucasians) but may present with acral lentiginous melanoma or mucosal melanoma. Mucosal melanomas account for 1.3 % of melanomas in whites and 11.8 % of all melanomas in blacks [11]. The age of occurrence in our case was 32 which is not consistent with the other reported cases nevertheless the age was above 20 as malignant melanoma is not common in patients below 20 years.

Ultra violet (UV) radiation plays a vital role in the physiopathology of cutaneous melanomas; oral mucosal melanomas usually appear in areas protected from UV radiation. Risk factors relative to the development of mucosal melanomas are unknown. Apparently there is no correlation to chemical, thermal, or physical events, and according to a study performed at the Dr. Manuel Gea Gonzalez Hospital (by Aguilar et al.) no relationship was found between mucosal melanoma and human papilloma virus (HPV) infections [9]. The absence of any risk factors linking to the etiology of mucosal melanoma in our patient was consistent with current belief that most oral melanomas emerge de novo.

Oral mucosal melanomas (OMM) are indolent and asymptomatic until the condition worsens. Most people do not inspect their oral mucosa properly until swelling, dental mobility, or bleeding occurs. Early lesions appear as a variable size pigmented macules whereas long lasting lesions can be nodular or pedunculated, pigments vary from dark brown to blue, gray purple or black. Nevertheless, it is common to find white or red macules, especially in swollen lesions.

However, lighter and near normal tissue color (amelanotic) can occur and up to one-third of oral mucosal melanomas may be amelanotic [7, 12]. For the first time, our patient presented with black papules of various sizes on the left vermilion along with brownish discoloration of skin above vermilion. The patient was aware of the changes in his lip since 4 years but reported so late. The progression of the lesion is very slow; it’s a very late presentation when the tumor has been allowed to grow slowly and locally for a long time. Had the patient presented earlier, such aggressive excision would not have been necessary. This shows that how the patient behavior has helped in the progression of the melanoma. The patient was cautioned against the chances of local recurrence and advised to maintain regular follow-up. The patient ignored the follow-up because of its asymptomatic behavior. The patient presented with pigmented macules and fibrotic mass on the upper lip during the time of recurrence.

Since the clinical manifestation of oral melanoma varies as an unevenly shaped macule, plaque or mass, well circumscribed or diffused and there is no distinct appearance to oral melanoma, the differential diagnosis is extensive. It can include Addison disease, blue nevus, lentigines, Kaposi sarcoma, oral nevus, amalgam tattoos, mucosal melanotic macule, Peutz–Jeghers syndrome, smoker’s melanosis and physiological pigmentation [9]. Oral amelanotic melanomas are rare and the prognosis is poorer than that of pigmented melanomas because of delay in establishing the correct diagnosis and the initiation of treatment. The differential diagnosis of amelanotic melanoma includes poorly differentiated carcinoma and lymphoma [13].

Initially, oral melanomas are typically asymptomatic; however, they can become painful with growth and expansion. Ulceration, bleeding, paresthesia, and ill-fitting prostheses are common complaints of patients presenting with late stage disease [7, 14, 15].

**Fig. 3:**
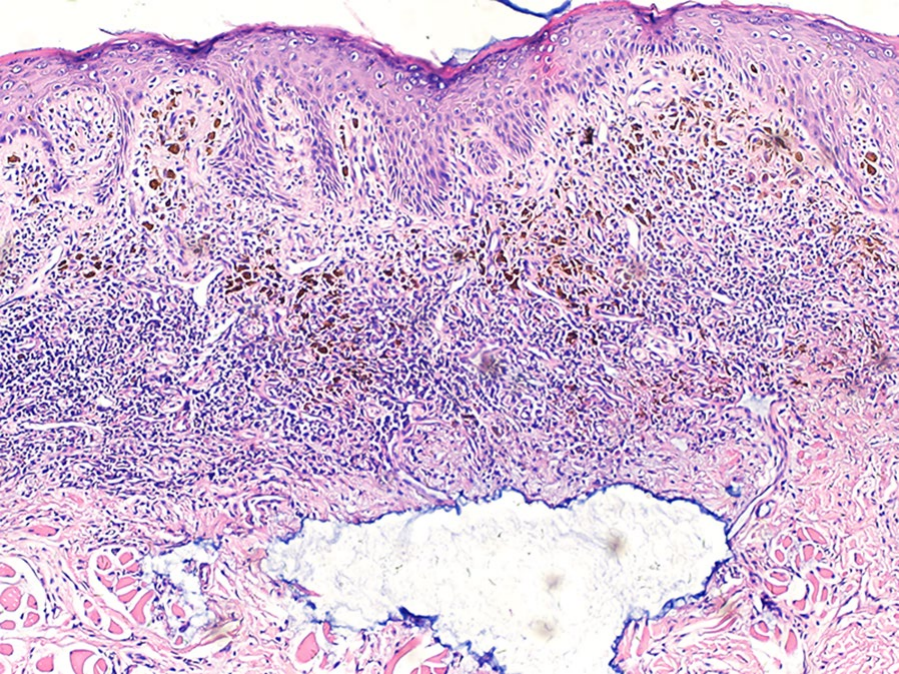
Epithelioid melanoma cells invasion into lamina propria, which are arranged in sheets and islands, containing large prominent nucleoli and melanin (×4)

**Fig. 4:**
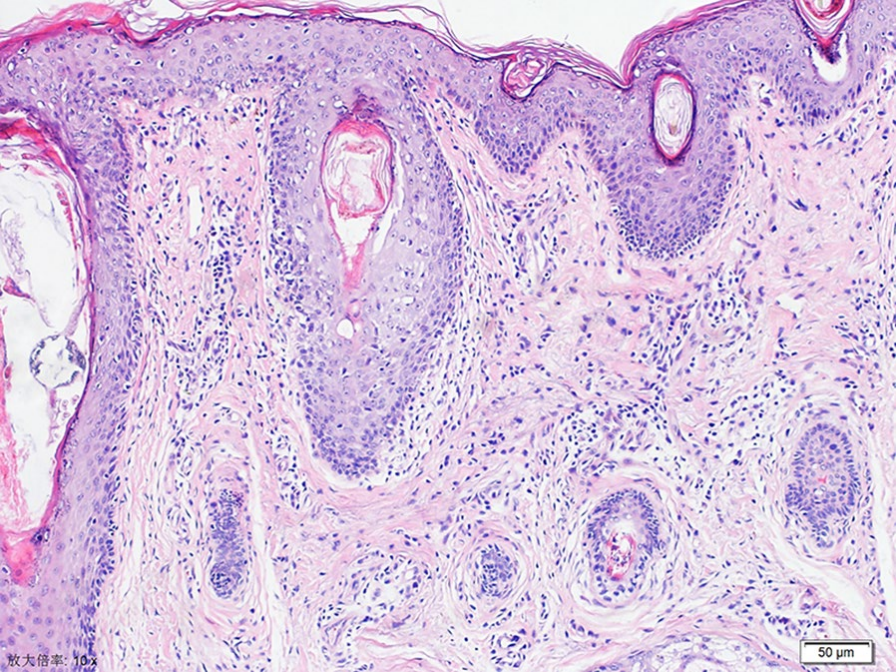
Intraepithelial hyperkeratosis, invasion of lamina propria by epithelioid melanocytes, which are forming nests and clumps (×10)

The definite diagnosis must be performed through histopathological study. The most important histopathologic finding is an epithelioid or fusiform (sarcomatose) or neural, melanocytic proliferation in asymmetric shape nest arrays. In the dermal epidermis junction, there is a predominance of individual cells with an abundant eosinophilic, clear cytoplasm, and melanin granules. They can have a large nucleolus, with prominent eosinophilic nucleoli and nuclear pseudo inclusions are found due to nuclear membrane irregularities. Necrosis and ulcerations are not unusual. Our patient had epithelioid melanoma cells invasion into lamina propria. Epithelioid melanocytes were arranged in sheets forming islands containing large prominent nucleoli and melanin (Fig. 3). The histological examination following recurrence showed intraepithelial hyperkeratosis, invasion of lamina propria by epithelioid melanocytes which were forming nests or clumps (Fig. 4). The histopathological differential diagnosis is extensive; therefore, in some occasions, immune-staining is required. Cells are positive for S-100, HMB-45, Melan-A, tyrosinase and microphthalmic-associated transcription factor (MITF) [9]. Amelanotic growths do not have melanin-pigmented tumor cells that vividly display Hematoxylin and Eosin staining, in which cases Immunohistochemistry is fundamental in establishing the final diagnosis [2].

Greene et al. [2] proposed three useful criteria in the diagnosis of primary oral melanoma. It includes:

1. The presence of clinical and microscopic tumor in oral mucosa.
2. The presence of junctional activity.
3. Inability to demonstrate any other primary site.

This patient fulfilled above three criteria for the diagnosis of primary oral melanoma and ruled out the possibility of metastasis from another primary melanoma.

Given the propensity for mucosal melanoma to disseminate and to exclude metastatic melanoma from a cutaneous primary, a basic metastatic workup should be considered. This workup includes serum lactate dehydrogenase, chest radiograph, and combined positron emission tomography/computed tomography scanning of the chest, abdomen, and pelvis [16]. Considering the fact, Chest X-ray and CT of chest, abdomen and pelvis were done in our patient to determine the extent of the disorder, local or regional involvement of lymphatic nodes and to rule out metastatic melanoma from a cutaneous primary.

Sentinel-node biopsy beneficial in cutaneous melanoma staging is less valuable in staging or treating oral melanoma, given they do not predict the tumor’s lymphatic drainage due to the existing anatomical ambiguity and as a result the erratic drainage does not allow for a consistent evaluation of how this method is used. There are not many studies regarding the role of sentinel lymph node biopsy in head and neck mucosal melanoma. In one study by Sta’rek et al. in 2006, they found that presence of microscopic metastatic focus in the sentinel lymph node was associated with an early hematogenous dissemination. Therefore, sentinel lymph node biopsy, which represents a potentially efficient staging tool, warrants further investigation [17].

The American Joint Committee on Cancer (AJCC), Cancer Staging Manual 7th edition (2010) includes a newly developed staging system for mucosal melanoma of head and neck [18]. The new staging criteria reflect the aggressive nature of head neck mucosal melanoma. The AJCC staging system for MM begins with stage T3 as the most limited form of disease. According to this system, our patient is Stage IVA. Mucosal melanoma tumor staging with negative lymph nodes were proposed by Prasad et al. [19] and Patel et al. [20]. Stage I is melanoma in situ (non-invasive), Stage II is the one invading the lamina propria and Stage III is the one invading deeper tissues. According to this staging system, our case falls in Stage II as it has invaded lamina propria. Survival average drops as stages progresses.

The Clark and Breslow classifications have not been validated as prognostic predictors in oral melanoma due to architectural differences between oral mucosa and skin. The oral mucosa is thinner than skin and lacks histological points of reference similar to the papillary and reticular dermis, nevertheless, some studies have compared oral melanomas with the acral lentiginous melanoma and with the cutaneous nodular melanoma [2]. Most authors use the classification of the Western Society of Teachers of Oral Pathology (WESTOP), which divides them into a relatively simple system according to its histopathological pattern as: (a) melanoma in situ, delimited to the epidermis and its junction with the connective tissue; (b) invasive melanomas, in which the neoplasia extends into the connective tissue and (c) melanomas with a combined pattern between invasive and in situ [21]. So far our case is concerned, the neoplastic cells extended into the connective tissues.

C-KIT is a key regulator of growth, differentiation, migration, and proliferation of melanocytes [22]. It has been shown to recruit and activate a number of intracellular signaling pathways implicated in tumor progression, such as the phosphoinositide 3-kinase/AKT, Src, mitogen-activated protein kinase, Janus kinase, signal transducers and activators of transcription, and phospholipase-C-g pathways [23]. Activating mutations in the c-*KIT* gene are detected in a significant number of patients with mucosal melanoma. The mitogen-activated protein kinase (MAPK) pathway (RAS/MEK/ERK) is a critical growth cascade in oral mucosal melanoma and it is the most common pathway described in oncogenic events during the progression of melanoma [24]. The MAPK pathway is downstream of the receptor tyrosine kinases, cytokines and G protein-coupled receptors, leading to cell growth, survival and differentiation. Molecules that participates in this signal transduction pathway are RAF (three isoforms ARAF, BRAF, CRAF) and RAS. RAS is encoded by the RAS gene, consisting of three isoforms HRAS, KRAS and NRAS. The intense RAS protein expression in both the in situ and invasive phases of oral mucosal melanoma (OMM) may suggest that RAS overexpression is necessary in OMM progression. A review of literature reports that 14 % of mucosal melanomas harbor activating c-KIT mutations; 5 % showed BRAF mutation and 14 % oncogenic mutations in NRAS, which is much lower than the reported BRAF prevalence (56–59 %) in cutaneous melanoma [25]. In addition, the MAPK pathway may be triggered by the activation of c-KIT, leading to the induction of signaling proteins, essentially stuck in the ‘on’ position, resulting in uncontrolled cell proliferation and survival [22]. Mutations in the c-KIT gene, along with overexpression of RAS in part, considered to be involved in the mechanism of development and progression of melanoma, have been identified in mucosal melanoma, suggesting c-KIT and RAS as a promising molecular target. Thus, drug therapies have been developed to inhibit these mutations, preventing tumor proliferation. The frequency of intense NRAS protein expression, BRAF and c-KIT activating mutations indicates that overlapping of molecular activities may occur in OMM progression posing a major concern in OMM therapy [26]. Such complex interactions of signal protein at multiple levels and with multiple pathways may require combinations of targeted therapies, rather than a single agent. Such tests for cKIT and RAS mutation as well as advanced targeted therapies are not available in our centre.

The best-validated targeted drugs in melanoma are the selective BRAF inhibitors vemurafenib (PLX4032, Zelboraf™) and dabrafenib (GSK2118436, Tafinlar™) as well as the LGX818 (Novartis) compound that appears to have the highest affinity for the catalytic domain of the kinase. All of them are relatively selective for their intended target V600E BRAF, with little cross-reactivity for wild-type BRAF and CRAF. These molecules selectively inhibit the growth of cells that harbour a V600 BRAF mutation. Vemurafenib and dabrafenib have both demonstrated impressive clinical efficacy with response rates in the region of 50 % in V600 BRAF mutated advanced melanoma. In contrast to BRAF mutated melanoma, the kinase inhibitor imatinib has proven efficacy in patients with advanced melanoma harbouring KIT mutations. KIT mutations are found at low frequencies (≤10 %) in melanomas arising from mucosal or acral lentiginous surfaces [27]. 50 % of patients who are treated with BRAF or MEK inhibitors have disease progression within 6–7 months after the initiation of treatment. Several mechanisms mediating resistance to BRAF inhibitors through MAPK reactivation have been described, including the up-regulation of bypass pathways mediated by cancer Osaka thyroid kinase (COT), development of de novo NRAS or MEK mutations, and dimerization or variant splicing of mutant BRAF V600. A study conducted by Keith T. Flaherty et al. in 2012 concluded that the combination of the BRAF inhibitor dabrafenib and the MEK inhibitor trametinib in patients with metastatic BRAF V600 melanoma, represents one strategy for delaying the emergence of this resistance mechanism (median duration of response for combination therapy 10.5 vs 5.6 months for dabrafenib monotherapy) [28].

Surgical resection when feasible remains the treatment of choice for oral melanomas. Adjunctive therapy (immuno/chemo/radio) is also often advocated. However, the literature reports no improvement with adjunctive therapies as it pertains to the overall survival rate [29, 30]. Recent reports supporting the use of biochemotherapy (combination of chemotherapy + interleukin 2/interferon) have been encouraging. Sun et al. found a significantly higher 5 year survival rate in patients treated with surgery and biochemotherapy when compared to those treated with surgery, chemotherapy, surgery and chemotherapy, or surgery and radiotherapy (58.4 vs 20.7 %). Our patient did not wish to undergo radiotherapy due to non-involvement of cervical lymph nodes.

Elective neck dissection was not performed in our patient as cervical lymphadenopathy was absent and also considering the fact that prophylactic lymph node dissection does not impact outcomes and is reserved for patients with clinically evident nodal involvement [16]. The enbloc resection decreases the chances the local recurrence rate, with little effect on metastasis and survival. Therapeutic neck dissection is to be done in cases of palpable lymph nodes but there is disagreement over neck dissection be done in absence of clinically palpable nodes [1].

Immunotherapy is useful in the treatment of melanoma at high risk for recurrence and for metastatic melanoma. Interleukin-2 (IL-2) was the first immunotherapy to be approved for metastatic melanoma (1998) and was approved on the basis of long-lasting complete response. Immunotherapy with BCG (Bacilli Calmette Guerin) which sometimes is used in patient with the intent of activating the host immune response has also been used but with little success. Other immunotherapeutic drug includes interferon and cimetidine, which when used together is believed to attack killer T cell and inhibit suppressor T cells which result in reduction of tumor size. Interferon injections have been of some benefits in patient with cutaneous and some metastatic melanoma, but the response to oral melanoma remains uncertain. Ipilimumab, an antibody that blocks the cytotoxic T-lymphocyte antigen-4 (CTLA-4) immune checkpoint, is approved by the United States Food and Drug Administration (FDA) in 2011 based on an overall survival (OS) advantage in patients with metastatic melanoma, however its efficacy in mucosal melanoma is not clear yet [31]. Programmed cell death-1 (PD-1), an immunoinhibitory receptor of the CD28 family, plays a major role in tumor immune escape. The interaction of PD-1 with its two ligands, B7-H1 and B7-DC (PD-L1 and PD-L2), occurs predominantly in peripheral tissues including the tumor microenvironment and leads to apoptosis and downregulation of T-cell effector function [32]. Monoclonal antibodies against PD1 and its ligand (PD-L1), the second generation immunomodulatory antibodies, displayed significant durable benefits in patients with MM [33]. Pembrolizumab and Nivolumab are the first and the second anti-PD-1 drug to receive accelerated approval in 2014 for demonstrating durable responses in patients whose disease has progressed following ipilimumab and, if BRAF V600 mutation positive, also a BRAF inhibitor.

Although previously melanoma was considered to be radioresistant, radiotherapy is now considered to be important adjuvant in achieving local control and may even has merit as primary therapeutic modality [34]. Furthermore, primary irradiation is considered as viable alternative to surgery for inoperable cases. It has also been used as an adjuvant treatment for recurrences, palliative treatment or postsurgical when the margins are doubtful.

While the recommended treatment is ablative surgery with tumor free margins and to a lesser extent, immunotherapy or radiotherapy, there is a recognized need for evidence based treatment protocol. Multimodality treatment may be more beneficial in the treatment of mucosal melanoma. It is apparent, however, that oral melanomas are much more aggressive than their cutaneous counterpart. The more aggressive behavior has been attributed to angioinvasion, anatomic relation that precludes adequate surgical removal, and delay in diagnosis, tendency to early ulceration owing to repeated trauma, which in turn may establish avenues for metastasis and higher rate of regional and systemic spread. Our case can be considered to be aggressive as there was recurrence following 3 and half years despite of tumor free margins, anatomical constraints due to esthetics and tendency to ulceration due to chances of trauma to lips. Literature also claims that patients with lesions under 2 mm in thickness have an important survival rate over those with lesions greater than 2 mm. Our patient having a lesion of over 2 mm thickness has a recurrence after 3 and half years with no distant metastasis. If lymphatic glands are affected prognosis drops down. Prognosis improves with early detection and total removal of lesion before it spreads. Eighty percent (80 %) of patients with oral mucosal melanoma have a local disease, 5–10 % of cases have neck and/or subclavian lymphatic node involvement. After complete removal, 10–20 % regional relapses have been reported with a 10–25 % 5 years survival rate [9].

## Conclusion

Oral melanoma is a rarer lesion which basically originates from the malignant transformation of melanocytes. There are no specific etiological factors identified for oral melanomas. So, clinician’s must be always vigilant to find these rare lesions, and pigmented lesions with growth potential should be submitted to biopsy to rule out malignancy. The patient should also be conscious enough to report pigmented lesions as soon as he/she is aware of it despite of it being asymptomatic so as to avoid aggressive treatment. Vigilant comprehensive analysis of published cases and recognition of new ones may be helpful in establishing definite classification and proposing clinical features that would facilitate its early diagnosis, as a prerequisite for timely treatment and better prognosis of this rare pathology.

## Consent

Written informed consent was obtained from the patient for publication of this Case Report and any accompanying images.

## Abbreviations

AJCC: American Joint Committee on Cancer
CT: computerized/computed tomography
WESTOP: Western Society of Teachers of Oral Pathology
MM: mucosal melanoma
OMM: oral mucosal melanoma
MAPK: mitogen activated protein kinase
PD-1: programmed cell death-1
